# The dCas9-based genome editing in *Plasmodium yoelii*

**DOI:** 10.1128/msphere.00095-24

**Published:** 2024-02-27

**Authors:** Chao Zhang, Shijie Yang, Elvis Quansah, Ziyu Zhang, Weiran Da, Bingjie Wang

**Affiliations:** 1 Department of Microbiology and Parasitology, Anhui Key Laboratory of Zoonoses, School of Basic Medical Sciences, Anhui Medical University, Hefei, China; 2 The Second Clinical Medical College, Anhui Medical University, Hefei, China; 3 The First Clinical Medical College, Anhui Medical University, Hefei, China; University at Buffalo, Buffalo, New York, USA

**Keywords:** malaria, *Plasmodium *parasites, CRISPR-Cas9, microbial single-strand annealing proteins, dCas9-SSAP

## Abstract

Genetic editing is a powerful tool for functional characterization of genes in various organisms. With its simplicity and specificity, the CRISPR-Cas9 technology has become a popular editing tool, which introduces site-specific DNA double-strand breaks (DSBs), and then leverages the endogenous repair pathway for DSB repair *via* homology-directed repair (HDR) or the more error-prone non-homologous end joining (NHEJ) pathways. However, in the *Plasmodium* parasites, the lack of a typical NHEJ pathway selects for DSB repair through the HDR pathway when a homologous DNA template is available. The AT-rich nature of the *Plasmodium* genome exacerbates this drawback by making it difficult to clone longer homologous repair DNA templates. To circumvent these challenges, we adopted the hybrid catalytically inactive Cas9 (dCas9)-microbial single-stranded annealing proteins (SSAP) editor to the *Plasmodium* genome. In *Plasmodium yoelii*, we demonstrated the use of the dCas9-SSAP, as the cleavage-free gene editor, by targeted gene deletion and gene tagging, even using shorter homologous DNA templates. This dCas9-SSAP method with a shorter DNA template, which did not require DSBs, independent of HDR and NHEJ, would be a great addition to the existing genetic toolbox and could be deployed for the functional characterization of genes in *Plasmodium*, contributing to improving the ability of the malaria research community in characterizing more than half of genes with unknown functions.

**IMPORTANCE:**

Malaria caused by *Plasmodium* parasites infection remains a serious threat to human health, with an estimated 249 million malaria cases and 608,000 deaths worldwide in 2022, according to the latest report from the World Health Organization (WHO). Here, we demonstrated the use of dCas9-single-stranded annealing protein, as the cleavage-free gene editor in *Plasmodium yoelii*, by targeted deletion and gene tagging, even using shorter homologous DNA templates. This method with a shorter DNA template, which did not require DSBs, independent of HDR and NHEJ, showing the potential significance in greatly improving our ability to elucidate gene functions, would contribute to assisting the malaria research community in deciphering more than half of genes with unknown functions to identify new drug and vaccine targets.

## INTRODUCTION

Malaria, as an ancient disease, caused by *Plasmodium* parasites infection, still poses a serious threat to the health of all humanity. According to the latest report from the World Health Organization (WHO), it is estimated that there will be 249 million malaria cases and 608,000 deaths worldwide in 2022 (1). The emergence and spread of antimalarial resistance have made the eradication goal a difficult task (2). This necessitates the need to explore new drug and vaccine targets.

The editing/manipulation of the parasite’s genome is a useful technology that allows for functional screening and characterization of potential drug and vaccine targets (3). The CRISPR-Cas9 (Clustered Regularly Interspaced Short Palindromic Repeats and CRISPR-associated protein 9) technology, was first applied to Plasmodium in 2014 and has since become a popular editing tool for *Plasmodium* (4–9). Mechanistically, the Cas9 generates a double-strand break (DSB) in the genome and leverages the endogenous repair pathway for DSB repair *via* homology-directed repair (HDR) or the more error-prone non-homologous end joining (NHEJ) pathways (10, 11). However, in malaria parasites, the lack of a typical NHEJ pathway selects for DSB repair through the HDR pathway when a homologous DNA template is available. Nonetheless, in *Plasmodium*, cloning a long homologous DNA template is arduous due to the parasite’s AT-rich genome (12). Against these challenges, it is necessary to develop a genome editing method that does not require DSBs, independent of HDR and NHEJ, while reducing the length of DNA templates makes cloning AT-rich DNA sequences easier.

Recently, a cleavage-free hybrid technology, comprising a catalytically inactive Cas9 (dCas9) coupled to microbial single-stranded annealing proteins (SSAPs), has emerged as a promising alternative for genomic editing with low off-targets and reduced unintended genomic scars. The SSAPs can bind to DNA substrates and then promote recombination between the genomic and donor homologous DNA sequences without creating DSBs (13). The dCas9-SSAP editor has been efficaciously used to knock in DNA sequences with different lengths of DNA templates in mammalian cell lines (14). However, whether the dCas9-SSAP could be used to edit the genome of malaria parasites has not been determined. Here, we developed and reported the use of dCas9-SSAP in *Plasmodium yoelii* by targeted gene deletion and gene tagging, even using relatively shorter homologous DNA templates.

## RESULTS

**Fig 1:**
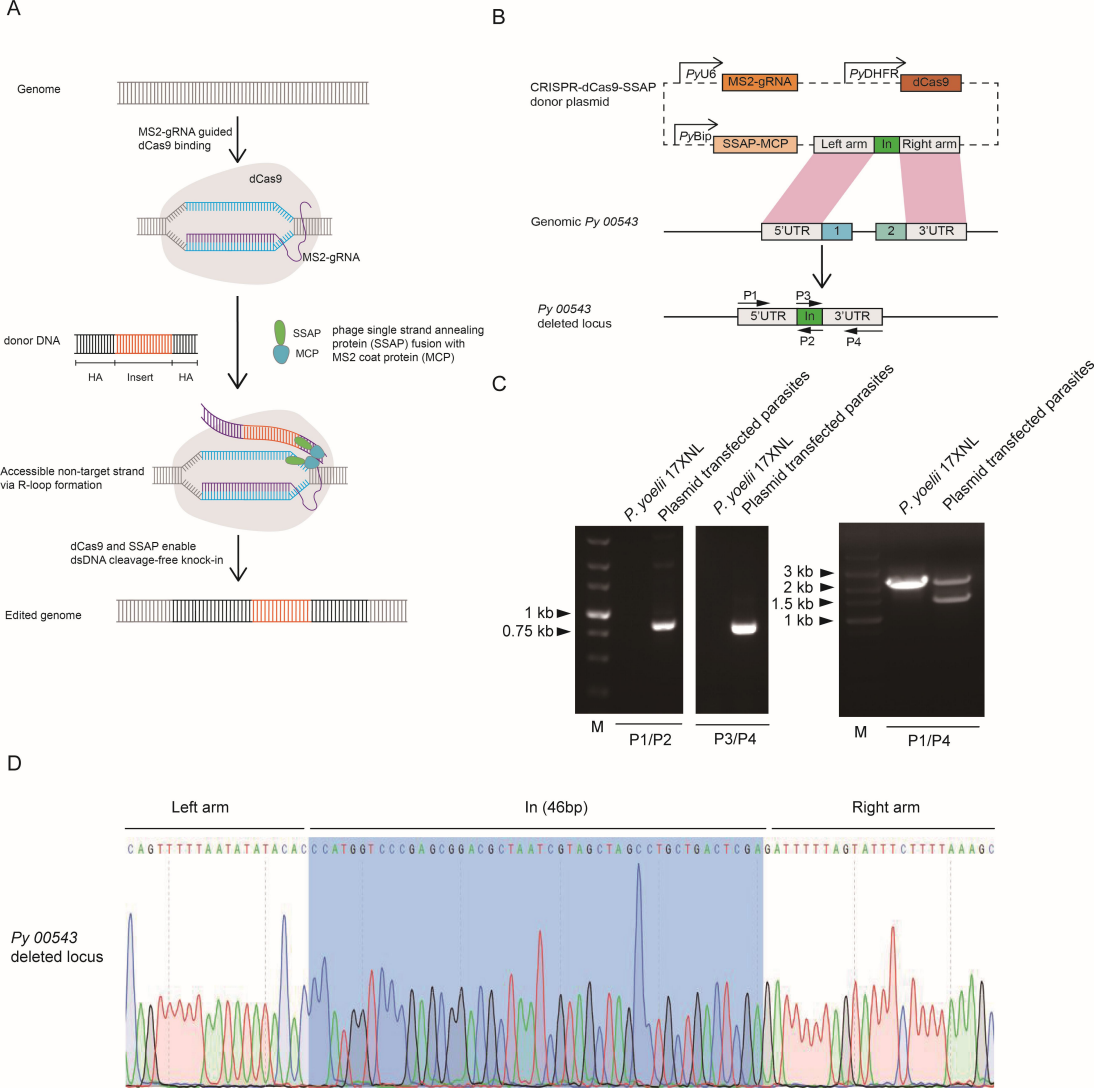
The model of microbial single-stranded annealing proteins (SSAPs) with CRISPR genome targeting and the assay to disrupt the gene in *Plasmodium yoelii*. (**A**) Schematic showing the model of SSAPs with CRISPR genome targeting. Note the sizes of elements were not drawn to scale. (**B**) Schematic construct for the dCas9-SSAP-mediated deletion of *Py05543* gene. The plasmid contained dCas9, gRNA bearing MS2 stem-loops, SSAP RecT, which was fused to the MS2 coat protein (MCP), and donor template. The DNA inserted (In) between the left and right arms was added. Exons 1 to 2 were indicated. The positions and directions of primers P1 to P4 were indicated by the small black arrows. (**C**) PCR identification for targeted *Py05543* deletion. (**D**) DNA sequencing confirmed the deletion in the *Py05543* gene. The partial nucleotide sequences of the left and right arms upstream and downstream of the *Py05543* gene, respectively, and the 46 bp DNA insert between the left and right arm sequences were shown.

To utilize SSAPs for genome editing in malaria parasites, we selected and modified *Escherichia coli Rac prophage* RecT (RecT), with the highest efficiency, as previously reported in mammalian cells (15). We cloned SSAP RecT into the expression plasmid of *Plasmodium* and structurally linked it to the CRISPR-dCas9 through RNA aptamers. As shown in Fig. 1A, the modularity of aptamer strategy was employed: gRNA carrying the MS2 stem-loops, SSAP RecT fused with the MS2 coat protein (MCP), and dCas9 that cannot cut but can bind to DNA to unwind and form an R-loop, making the non-target strand putatively accessible for SSAP-stimulated homologous recombination (15).

To measure the gene editing activity of dCas9-SSAP in malaria parasites, we first attempted to delete the parasite gene *Py05543*, which is nonessential for the growth of blood stages (16). The plasmid pDEF-dCas9-MCP-RecT-*Py05543* containing a 46 bp tag DNA ﬂanked by two homologous regions of *Py05543* (403 bp of the 5′-ﬂanking region and 499 bp of the 3′-ﬂanking region) was constructed (Fig. 1B). PCR analysis of genomic DNA from parental strain and plasmid-transfected parasites indicated the parasites with *Py05543* deletion were successfully established (Fig. 1C). PCR products from plasmid-transfected parasites were puriﬁed and sequenced directly to confirm the targeted deletion in the *Py05543* gene (Fig. 1D).

**Fig 2:**
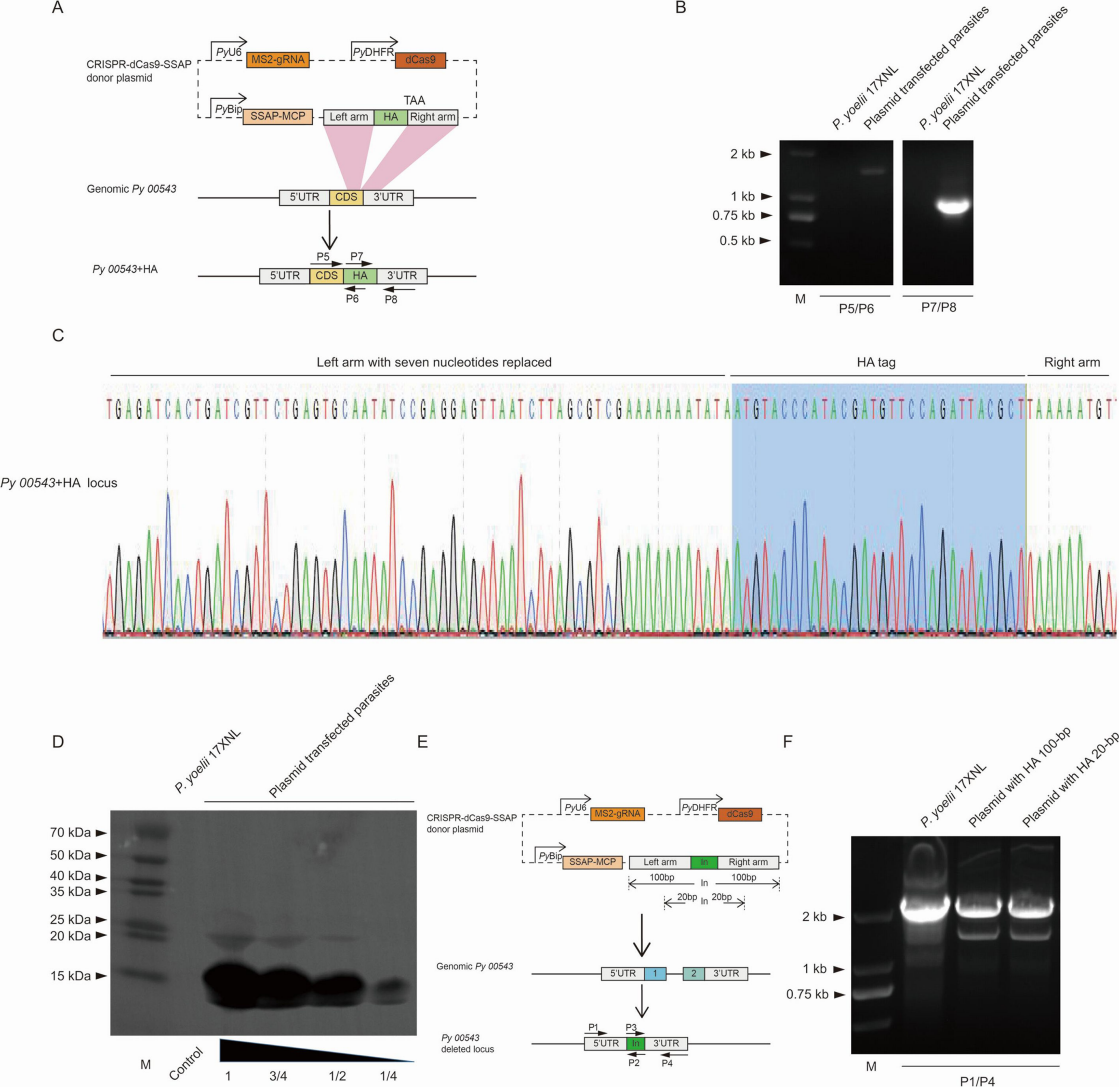
The assays for tagging the endogenous gene and validation across donor designs with shorter DNA templates in *Plasmodium yoelii*. (**A**) Schematic construct for tagging *Py05543* gene with HA tag. The plasmid contained sgRNA expression cassettes and donor templates targeting the C-terminal part of the coding sequence (CDS) of the *Py05543* gene. The directions and positions of primers P5 to P8 were indicated by the small black arrows. (**B**) PCR detection of 5′ and 3′ integration of HA tag into the *P. yoelii* 17XNL genome. (**C**) DNA sequencing conﬁrmation of HA-tagged *Py05543* gene. (**D**) The protein expression of the *Py05543* gene was detected using immunoblotting with anti-HA antibody. *P. yoelii* 17XNL with no transfection was used as a control. The positions of molecular mass markers (in kilodaltons) were indicated to the left of the blots. (**E**) Schematic construct for deleting *Py05543* gene with shorter DNA templates. The DNA inserted (In) between the left and right arms of shorter DNA templates was added. The positions and directions of primers P1 to P4 were indicated by the small black arrows. (**F**) PCR identification for targeted *Py05543* deletion with shorter DNA templates.

Next, we used dCas9-SSAP to append a C-terminal HA epitope tag to *Py05543*. For this, the plasmid pDEF-dCas9-MCP-RecT-*Py05543*-HA containing a 375 bp C-terminal region of the *Py05543* gene followed by the HA tag and a 366 bp 3′ untranslated region (3′UTR) of the *Py05543* gene was constructed (Fig. 2A). To prevent binding to the target site of the donor template after integration, seven nucleotides in the sgRNA binding site were replaced as a shield mutation (4). The parasites with integration of donor template into the 3′ end of the *Py05543* gene 5 days after transfection were successfully established (Fig. 2B). PCR products from plasmid-transfected parasites were puriﬁed and sequenced directly to confirm the gene tagging in the *Py05543* gene (Fig. 2C). As expected, immunoblot detection of the HA tag fused to the *Py05543* gene revealed an expected 16 kDa product, suggesting protein expression of the *Py05543* gene (Fig. 2D).

The high efﬁciency of gene modiﬁcation using short template sequences would benefit studies of AT-rich genomes from the malaria parasite because long AT-rich DNA sequences are often unstable in *Escherichia coli* and difficult to be cloned into plasmids. We measured the gene modiﬁcation using dCas9-SSAP with shorter DNA templates. The plasmid pDEF-dCas9-MCP-RecT-*Py05543* containing a 46 bp tag DNA ﬂanked by two homologous regions of *Py05543* (100 bp, 20 bp of the 5′-ﬂanking region and the 3′-ﬂanking region, respectively) was constructed (Fig. 2E). PCR analysis of genomic DNA from parental strain and plasmid-transfected parasites indicated the parasites with *Py05543* deletion using 100 bp or 20 bp lengths of DNA template were successfully established (Fig. 2F).

## DISCUSSION

Here, we have developed a method based on dCas9-SSAP and successfully adapted it to *Plasmodium yoelii*, by effectively achieving the targeted gene deletion and gene tagging, even using relatively shorter DNA templates. It has been estimated that over one-third of the *Plasmodium* encoding genes remain uncharacterized and this situation is likely not to change if new effective functional characteristics tools are not added (17–19). Our results showed the practicability of dCas9-SSAP in *Plasmodium yoelii* and thus provided an additional tool to the existing toolbox for elucidating *Plasmodium* gene functions.

However, to expand the application scopes of the dCas9-SSAP method in malaria parasites, the following aspects need further optimization in the future. First, although we successfully achieved effective gene editing, using the dCas9-SSAP method, the designing and selection of a sgRNA presented a formidable technical challenge. Specifically, editing efficiencies of the sgRNA differed depending on the target site. This limitation was fueled by the AT content in the malaria parasite genome. Second, the size (more than 12 kb) of the plasmids used in this study was relatively large and inherently unstable during cloning. In light of this, optimizing and selecting compact plasmids, such as using SaCas9 with more than 1 kb shorter size than Cas9, might be required in future attempts (20). Finally, it remains to be determined whether the dCas9-SSAP could work in *P. falciparum*, to address the existing challenges associated with CRISPR-Cas9. For example, the accidental editing of the CRISPR-Cas9 genome in subtelomeric regions also resulted in the loss of all DNA between the Cas9 cleavage site and the chromosome end, as well as an increase in telomere repeat sequences (21). In this regard, efforts are ongoing to establish this technique in *P. falciparum* by our team.

To our knowledge, the dCas9-SSAP has not yet been adapted to *Plasmodium* nor any apicomplexan. In this observation, we have for the first time demonstrated the dCas9-SSAP could work in *Plasmodium* parasites. Given the dCas9-SSAP method did not require DSBs, independent of HDR and NHEJ, it would help circumvent pitfalls such as off-target cleavage and genomic loss that are associated with DBSs. In addition, gene modiﬁcation using the dCas9-SSAP method with these shorter DNA templates would benefit studies of AT-rich genomes from the malaria parasite. The previous Walker and Lindner’s work in *P. yoelii* demonstrated that a short DNA donor is sufficient for ribozyme-mediated CRISPR-Cas9 gene editing (22). However, the dCas9-SSAP method and Walker and Lindner’s method have different mechanisms of action, showing different advantages, and would together contribute to assisting the malaria research community in deciphering more than half of genes with unknown functions to identify new drug and vaccine targets.

## MATERIALS AND METHODS

### Experimental animals and parasite lines

All animal treatments followed the guidelines for the care and use of experimental animals approved by the Animal Experiment Committee of Anhui Medical University. All mice were purchased and housed in the Animal Care Center of Anhui Medical University and kept at room temperature under a 12 h light/dark cycle at a constant relative humidity of 45%. All transgenic parasites were generated from the *P. yoelii* 17XNL strain, a gift from Professor Xiaoping Chen (Guangzhou Institutes of Biomedicine and Health, Chinese Academy of Sciences, China). The parasites were propagated in ICR (Institute of Cancer Research) mice (female, 5 to 6 weeks old).

### Plasmid construction

The guide RNA (gRNA) was designed upstream of the protospacer-adjacent motif (PAM), using the online CRISPR guide RNA/DNA design tool for eukaryotic pathogens (EuPaGDT; http://grna.ctegd.uga.edu), and a pair of complementary oligonucleotides were synthesized for each target site (Sangon Biotech, China). dCas9 nuclease coding fragment was ampliﬁed from pdCas9-DNMT3A-EGFP (Addgene Plasmid #71666) and replaced Cas9 nuclease coding fragment of the pDEF-SpCas9GFP (Addgene Plasmid #129523), then MCP-RecT coding fragment ampliﬁed from Addgene Plasmid #164803 and MS2-BB_BbsI fragment ampliﬁed from Addgene Plasmid #164802 were inserted, and finally the plasmid pDEF-dCas9-MCP-RecT-MS2-BB_BbsI was constructed.

To generate the dCas9-SSAP plasmid for deleting gene *Py*05543 (Gene ID in PlasmoDB database), the 5′- and 3′-ﬂanking genomic regions (403 and 499 bp) of the target genes were amplified as left and right homologous arms, respectively, using gene-specific primers. The left and right arms were inserted into the linker site in the pDEF-dCas9-MCP-RecT-MS2-BB_BbsI plasmid. A 46 bp DNA fragment was inserted between the left and right arms as a template for designing primers.

To generate the dCas9-SSAP plasmid for tagging gene *Py*05543 with HA tags, we ﬁrst ampliﬁed the C-terminal part (375 bp) of the coding region as the left arm and 366 bp from the 3′ UTR region following the translation stop codon as the right arm using the primers. A DNA fragment encoding the HA tag was inserted between the left and right arms in the frame with the gene of interest. One sgRNA was designed to target the site close to the C-terminal part of the coding region. To avoid binding of sgRNA to the target site in the left arm of the donor template after integration, seven nucleotides in the sgRNA binding site were replaced as a shield mutation.

To generate the dCas9-SSAP plasmid with shorter DNA templates in the gene *Py*05543, considering the high TA content in the malaria parasite genome, the 5′- and 3′-ﬂanking genomic regions (100 bp and 20 bp) of the target genes were chosen and synthesized as left and right homologous arms, respectively. The left and right arms were inserted into the linker site in the pDEF-dCas9-MCP-RecT-MS2-BB_BbsI plasmid. A 46 bp DNA fragment was inserted between the left and right arms as a template for designing primers. The designed oligonucleotides and gene-specific primers used in this study are shown in Table 1. All the plasmids constructed in this study will be shared with other researchers on request.

**TABLE 1:** The oligonucleotides and gene-specific primers

Name	Sequence (5′ to 3′)	Notes
The assay to disrupt the *Py05543* gene		
gRNA1 Forward	TATTGTTTGCTAGAGTAAAAACGCA	Targeting *Py*05543 locus
gRNA1 Reverse	AAACTGCGTTTTTACTCTAGCAAAC	
P2-1	GCGCAAGCTTGTAGCTTAATTATTGCTTATTC	Left arm for recombination, targeting the inserted sequence and not the genome
P2	GCTAGCTACGATTAGCGTCCGCGGGGACCATGGGTGTATATATTAAAAACTGTTTCC	
P3	GCGGACGCTAATCGTAGCTAGCCTGCTGACTCGAGATTTTTAGTATTTCTTTTAAAG	Right arm for recombination, targeting the inserted sequence and not the genome
P3-1	GCGCCTTAAGCAATGTTTTAACATCACAACTATATGC	
P1	GATAACTTTGTTGATTTAAAACTCTTTTC	Identification for recombination events, targeting the genome, and not the inserted sequence
P4	TTGAGAATAACCTCTTATTTCTATATATG	
The assay for tagging of *Py05543* gene with HA tag		
gRNA2 Forward	GCGTTCTGAATGTAACATAAG	Targeting *Py05543* locus
gRNA2 Reverse	CTTATGTTACATTCAGAACGC	
HA -La-F	GAGTTGTTAAACAAAAAAATAACAG	Left arm for recombination
HA -La-R1	TTCGACGCTAAGATTAACTCCTCGGATATTGCACTCAGAACGATCAGTGATCTCAG	
HA -La-R2	GCGTAATCTGGAACATCGTATGGGTACATTATATTTTTTTCGACGCTAAGATTAACTCC	
HA-Ra-F	ATGTACCCATACGATGTTCCAGATTACGCTTAAAAATGTTTGCATGC	Right arm for recombination
HA-Ra-R	AACTTAAACTTTTAACACAATTTTGCT	
P5	GTAGCTTAATTATTGCTTATTC	Identification for recombination events; P5 and P8 target the genome and not the inserted sequence; P6 and P7 target the inserted sequence and not the genome
P6	AGCGTAATCTGGAACATCGTATGGGTAC	
P7	ATGTACCCATACGATGTTCCAGATTACG	
P8	CAATGTTTTAACATCACAACTATATGC	
The assay for validation across donor designs with shorter DNA templates		
gRNA1 Forward	TATTGTTTGCTAGAGTAAAAACGCA	Targeting *Py*05543 locus
gRNA1 Reverse	AAACTGCGTTTTTACTCTAGCAAAC	
KO100-F	AAATTTTACTGATATCTTATTGATAATC	100 bp of left arm and right arm for recombination
KO100-R	GTATGTATAAAAATATAATAGAATTGGAATG	
KO20-F	ACAGTTTTTAATATATACAC	20 bp of left arm and right arm for recombination
KO20-R	TTAAAAGAAATACTAAAAATCTC	
P1	GATAACTTTGTTGATTTAAAACTCTTTTC	Identification for recombination events, targeting the genome, and not the inserted sequence
P4	TTGAGAATAACCTCTTATTTCTATATATG	

### Parasite transfection

Transgenic *P. yoelii* parasites were generated as described previously (1, 2). Briefly, about 1 × 10^7^ purified schizonts from infected red blood cells (iRBCs) were electroporated with 5 µg circular plasmid DNA using Lonza Nucleofector with the U-033 program. Transfected parasites were immediately injected intravenously into a naïve ICR mouse, and then selected with pyrimethamine (6 µg/mL) in drinking water, which was initiated 24 h post-infection. Pyrimethamine-resistant parasites usually appear 5 to 7 days after injection.

### DNA analysis

According to the previously reported method (7, 16), blood cell samples were collected from the orbital sinus of infected mice and lysed using 1% saponin in phosphate-buffered saline (PBS) to release parasites. These parasites were collected through centrifugation with 12,000 rpm for 3 min, and then the parasite genomic DNAs were isolated using DNeasy Blood kits (QIAGEN), as the template for PCR amplification (TaKaRa). The gene deletion and gene tagging were conﬁrmed by genotyping PCR using two pairs of primers to detect integrations. All PCR products were puriﬁed and sequenced directly (Sangon Biotech., China). PCR-specific primers used in this study are shown in Table 1.

### Western blotting

Total proteins extracted from parasite pellets were separated on 4 to 20% SDS-PAGE gels (Sangon Biotech., China) and transferred to polyvinylidene diﬂuoride (PVDF) membranes (Millipore), which were incubated with the blocking buffer [PBS with 3% bovine serum albumin (BSA)] for 1 h at room temperature and then incubated at 4°C overnight with anti-HA (mouse; 1:1,000; Beyotime Biotech., China). After three washes, the horseradish peroxidase-conjugated goat anti-mouse antibody (Beyotime Biotech., China) was incubated with PVDF membranes for 2 h at room temperature, and then after three washes, the enhanced chemiluminescence (ECL) kit (Beyotime Biotech., China) was used for detection.
